# *QuickStats:* Percentage* of Adults Aged ≥20 Years Reporting Depressive Symptoms^†^ in the Past 2 Weeks, by Sex — National Health and Nutrition Examination Survey, United States, 2013–2016

**DOI:** 10.15585/mmwr.mm6709a5

**Published:** 2018-03-09

**Figure:**
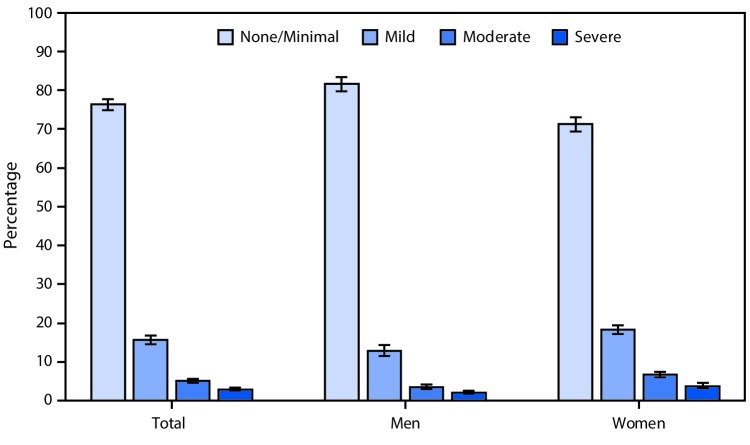
During 2013–2016, 76.3% of adults aged ≥20 years had no or minimal depressive symptoms, 15.6% had mild symptoms, 5.1% had moderate symptoms, and 2.9% had severe depressive symptoms. A lower percentage of women than men had no or minimal depressive symptoms (71.3% versus 81.6%), but a higher percentage of women than men had mild (18.3% versus 12.8%), moderate (6.7% versus 3.4%), or severe (3.7% versus 2.1%) symptoms.

**Source:** NCHS Data Brief No. 303. https://www.cdc.gov/nchs/data/databriefs/db303.pdf.

